# The hindgut microbiome contributes to host oxidative stress in postpartum dairy cows by affecting glutathione synthesis process

**DOI:** 10.1186/s40168-023-01535-9

**Published:** 2023-04-22

**Authors:** Fengfei Gu, Senlin Zhu, Jinxiu Hou, Yifan Tang, Jian-Xin Liu, Qingbiao Xu, Hui-Zeng Sun

**Affiliations:** 1 Institute of Dairy Science, College of Animal Sciences, Zhejiang University, Hangzhou, 310058 China; 2 Ministry of Education Key Laboratory of Molecular Animal Nutrition, Zhejiang University, Hangzhou, 310058 China; 3 College of Animal Sciences and Technology, Huazhong Agricultural University, Wuhan, 430070 China; 4 Ministry of Education Innovation Team of Development and Function of Animal Digestive System, Zhejiang University, Hangzhou, 310058 China

**Keywords:** Dairy cows, Fecal microbiota transplantation, Glutathione synthesis, Multiomics, Oxidative stress, Transition period

## Abstract

**Background:**

Dairy cows are susceptible to postpartum systemic oxidative stress (OS), which leads to significant production loss and metabolic disorders. The gut microbiota has been linked to host health and stress levels. However, to what extent the gut microbiota is associated with postpartum OS remains unknown. In this study, the contribution of the fecal microbiota to postpartum systemic OS and its underlying mechanisms were investigated by integrating 16S rRNA gene sequencing, metagenomics, and metabolomics in postpartum dairy cattle and by transplanting fecal microbiota from cattle to mice.

**Results:**

A strong link was found between fecal microbial composition and postpartum OS, with an explainability of 43.1%. A total of 17 significantly differential bacterial genera and 19 species were identified between cows with high (HOS) and low OS (LOS). Among them, 9 genera and 16 species showed significant negative correlations with OS, and *Marasmitruncus* and *Ruminococcus_sp._CAG:724* had the strongest correlations. The microbial functional analysis showed that the fecal microbial metabolism of glutamine, glutamate, glycine, and cysteine involved in glutathione synthesis was lower in HOS cows. Moreover, 58 significantly different metabolites were identified between HOS and LOS cows, and of these metabolites, 19 were produced from microbiota or cometabolism of microbiota and host. Furthermore, these microbial metabolites were enriched in the metabolism of glutamine, glutamate, glycine, and cysteine. The mice gavaged with HOS fecal microbiota had significantly higher OS and lower plasma glutathione peroxidase and glutathione content than those orally administered saline or LOS fecal microbiota.

**Conclusions:**

Integrated results suggest that the fecal microbiota is responsible for OS and that lower glutathione production plays a causative role in HOS. These findings provide novel insights into the mechanisms of postpartum OS and potential regulatory strategies to alleviate OS in dairy cows.

Video Abstract

**Supplementary Information:**

The online version contains supplementary material available at 10.1186/s40168-023-01535-9.

## Background

The transition period (3 weeks before and after parturition) is a critical stage of dairy cows; this is a period in which a majority of cows easily suffer from various diseases [1]. Oxidative stress (OS) is the most commonly observed phenomenon in the postpartum stage and contributes to multiple postpartum diseases or disorders, such as ketosis, fatty liver, and mastitis, in dairy cattle [2]. Thus, it is very important to alleviate postpartum OS for dairy cattle health and lactation performances. Postpartum OS in dairy cows is a complex interplay of multiple biological processes in different tissues, including uterine involution, onset of copious milk synthesis and secretion, adipose mobilization, and liver hypermetabolism [3]. Therefore, systemic OS should be reflected and evaluated by holistic parameters. The blood OS index (OSI), calculated based on total oxidative status and antioxidant capacity, can be used to assess the degree of OS [4, 5] and is regarded as a new tool to evaluate the redox status in perinatal dairy cows [6].

As the “second genome,” the gut microbiota has a substantial impact on various physiological functions, including metabolism and health of the host [7–9]. OS is one of the most vital mechanisms for diseases and is strongly associated with a dysfunctional gut microbiome [8–10]. Furthermore, increasing evidence has shown that gut microbial dysbiosis can cause minor to severe nuisances in local organs, such as the liver [11], gut [12], and brain [13], which in turn lead to systemic OS. In addition, the composition and richness of the gut microbiome change dramatically during the perinatal period [14]. Thus, we speculated that the gut microbiome may be related to postpartum systemic OS. However, the extent to which the gut microbiota contributes to systemic OS and the underlying mechanisms remain unclear. Previous studies suggest that gut microbial metabolites or gut microbial metabolic pattern variations may influence host physiology and behavior via multiple direct or indirect pathways [15–17]. Different omics approaches, especially 16S rRNA gene sequencing, metagenomics, and metabolomics, provide powerful tools to identify potential players in a high-throughput manner and to further elucidate microbial mechanisms. Integrating omics techniques yields a better understanding and clearer picture than single omics analysis [18, 19].

Thus, in this study, we aimed to answer two questions. Does the gut microbiome (composition and functions) contribute to OS (characterized by OSI) during the postpartum period in dairy cows? If so, what are the potential mechanisms? We integrated 16S rRNA gene sequencing, metagenomics, and metabolomics of cattle fecal samples at 7 days postpartum and fecal microbiota transplantation in mice to reveal the relationship between the gut microbiota and host systemic OS.

## Methods

### Animals and experimental design

Sixty-three healthy Chinese Holstein dairy cows (milk yield, 36.9 ± 8.04; parity, 2.75 ± 0.94; body condition score, 2.75 ± 0.35) without antibiotics or drug treatment were selected from a large cohort of 2000 dairy cows. These cows were raised in the same environment, for example, the same diet, water, and management. The diet composition is presented in Table S1.

### Sample collection

A total of 63 blood samples were collected into tubes containing an anticoagulant (EDTA vacutainer) from the coccygeal vein of cows 7 days after calving at 6:00 am. The samples were centrifuged at 3000 × g for 15 min at 4 °C to collect plasma. The plasma was frozen in liquid nitrogen and stored at − 80 °C for subsequent analysis. Feces were collected manually from the rectum of cows by using sterilized gloves before morning feeding. The feces were transferred to sterile 50 mL frozen storage tubes, quenched in liquid nitrogen immediately, and stored at − 80 °C for subsequent analysis.

### Plasma parameter measurement

The concentrations of glucose (#ZH2079T), total protein (#ZH2012G), blood urea nitrogen (#ZH2017S), nonesterified fatty acids (#ZH2045Z), β-hydroxybutyrate (#ZH2029T), cholesterol (#ZH2040Z), triglycerides (#ZH2039Z), albumin (#ZH2013G), superoxide dismutase (SOD, #ZH2058F), creatinine (#ZH2020S2), alanine aminotransferase (#ZH2001G), and aspartate aminotransferase (#ZH2002G) in plasma were measured using an AutoAnalyzer 7020 instrument (Hitachi High-Technologies Corporation, Tokyo, Japan) with commercial kits (Ningbo Medical System Biotechnology Co., Ltd., Ningbo, China). The concentrations of plasma catalase (CAT, #A007-1–1), glutathione (GSH, #A006-2–1), glutathione peroxidase (GSH-px, #A005-1–2), malondialdehyde (MDA, #A003-1–2), haptoglobin (HPT, #H136), amyloid (SAA, #H134), and total antioxidant capacity (T-AOC, #A015-2–1) were measured using commercial assay kits from Nanjing Jiancheng Bioengineering Institute (Nanjing, China) according to the manufacturer’s instructions. In addition, the plasma total oxidative status (TOS; #KC5100, Bensheim, Germany) was determined using commercial assay kits according to the manufacturer’s instructions. The OSI was calculated as the ratio of TOS to T-AOC. Each sample was replicated three times for the detection of the aforementioned parameters. The cows with lower (LOS, *n* = 9) and higher (HOS, *n* = 9) values but similar phenotypic characteristics, including milk yield, parity, and body scores were selected from the aforementioned 63 cows for subsequent exploration of the mechanisms of OS related to the fecal microbiome. LOS cows were regarded as the control reference in the current study. Body condition was scored following the method described by Edmonson et al. [20] using a 5-point scale (1 = thin, 5 = fat) at 3 time points (06:00, 14:00, and 20:00).

### Statistical analysis of plasma parameters

Statistical analysis and graphs of plasma parameters were generated using Prism (GraphPad Software Inc. 8.0, La Jolla, CA, USA). Student’s *t* test was used for comparisons between two groups, and ANOVA was used for comparisons among multiple groups. Significance was declared at *P* ≤ 0.05, and 0.05 < *P* ≤ 0.10 was considered a significant trend.

### DNA extraction and sequencing

The total fecal microbial DNA was extracted using the E.Z.N.A. ®Stool DNA Kit (#D4015, Omega, Inc., USA) for 16S rRNA gene and metagenome sequencing. A 1% agarose gel and NanoDrop 2000 spectrophotometer (Thermo Scientific, Wilmington, USA) were used for quality assessment and concentration measurement, respectively. The common primer pair (341F: 5′-CCTACGGGNGGCWGCAG-3′; 805R: 5′-GACTACHVGGGTATCTAATCC-3′) was used to amplify the V3–V4 region of the bacterial 16S rRNA gene. The PCR product was purified using AMPure XT Beads (Beckman Coulter Genomics, Danvers, MA, USA) and quantified using Qubit (Invitrogen, USA). Qualified PCR products were evaluated using an Agilent 2100 Bioanalyzer (Agilent, USA) and Illumina library quantitative kits (Kapa Biosciences, Woburn, MA, USA), which were further pooled together and sequenced on an Illumina NovaSeq PE250, provided by LC-Bio Technology Co., Ltd., Hangzhou, China.

### Data processing and 16S rRNA gene sequencing analysis

The raw sequence data were demultiplexed into sample paired-end fastq files using FLASH. Quality filtering was performed to obtain high-quality clean tags according to fqtrim (v0.94) [21]. Chimeric sequences were filtered using Vsearch software (v2.3.4) [22]. DADA2 [23] was applied for denoising and generating amplicon sequence variants (ASVs) of quality reads that were dereplicated at 100% sequence identity and clustered at 99% sequence identity. BLAST was used for sequence alignment, and taxonomic annotation was based on the SILVA (138 database) (https://www.arbsilva.de) [24]. Feature abundance was normalized using the relative abundance of each sample. Microbial taxa with relative abundances > 0.01% in more than 50% of the samples were used for downstream analysis. Alpha and beta diversities were calculated using QIIME2 [25]. Linear discriminant analysis effect size (LEfSe) was applied to analyze the significantly different bacteria between the HOS and LOS cows by using the Kruskal–Wallis test [26]. Significance was declared at *P* < 0.05 and a linear discriminant analysis (LDA) score > 2. For the reasons of failure of collecting enough amount/number of fecal samples or failed the quality control of DNA, there are 12 fecal samples of cows were missed. Therefore, we only sequenced for 51 fecal samples in current study. Furthermore, part of the microbiome data (*n* = 10) was same as the one used in our previous study [27].

### Co-occurrence network analysis

To understand microbial interactions in the gut, we constructed co-occurrence networks based on the relative abundance of genera in each group [28]. The genera correlation network within the HOS and LOS cows was analyzed separately by Spearman’s correlation coefficient in the R package Hmisc (v4.6.0). The significant correlation (p.adjust < 0.05, |rho|> 0.70) among different genera was visualized using Cytoscape v3.8.2 (http://www.cytoscape.org) [29]. Next, a comparison between the two network structures was conducted based on node closeness and shared correlations.

### Microbiability calculations

The linear mixed model (LMM) was performed using the R package Ime4qtl (v0.2.2) [30] and was used to estimate the OS variance explained by the gut microbiota:

$$Y=\mathrm{Kc }+\mathrm{ms }+\mathrm{u }+\mathrm{e},$$

where *y* is the phenotype OSI, and *c* is the vector of the fixed covariates, consisting of parity and milk yield. Fecal microbes in each animal are considered random effects, which follow the distribution ms ~ *N* (0, Mσ^2^_m_), where *M* is the relationship matrix constructed using the relative abundances of microbial genera based on the following formula:

$$M=\frac{1}{N}\sum_{a=1}^{N}\frac{(Aia -\overline{A }a)(Aja-\overline{A }a)}{{\upsigma }_{a}^{2}},$$

where $Aia$ and $Aja$ represent the relative abundance of the *a*th genus of individuals *i* and *j*, respectively; *u* is the intercept; and *e* is the residual effect. The phenotypic variance explained by the fecal microbial variance in dairy cows was estimated as the ratio of fecal microbial variance and phenotype variance ($\frac{{\upsigma }_{m}^{2}}{{\upsigma }_{p}^{2}}$), where ${\upsigma }_{p}^{2}$ represents the OSI variance, and ${\upsigma }_{m}^{2}$ represents the variance from gut microbiota calculated as a random effect. In the animal gut microbiome field, this part of variance is defined as “microbiability,” which is considered the extent to which fecal microbes contribute to the phenotype [17].

### Library construction, sequencing, and data processing of metagenomics analysis

Fecal samples of HOS and LOS cows (*n* = 18) were subjected to metagenome sequencing, and 10 microbiome datasets were used in our previous study [27]. The metagenomic DNA library was costructed using the TruSeq Nano DNA Library Preparation Kit-Set (#FC-121–4001, Illumina, USA). Metagenome libraries were sequenced on an Illumina NovaSeq6000 platform in a PE150 pattern. The quality control of each dataset was performed using cutadapt (v1.9) to remove sequencing adapters, low-quality reads (quality scores < 20), short reads (< 100 bp), and reads containing more than 5% “N” records by using the sliding-window algorithm method with fqtrim (v 0.94) [21]. The reads were aligned to the bovine genome (bosTau8 3.7, https://doi.org/10.18129/B9.bioc.BSgenome.Btaurus.UCSC.bosTau8) by using bowtie (V2.2) to filter the host DNA [31]. The filter reads were *assembled *de novo for each sample using IDBA-UD (v1.1.1) [32]. MetaGeneMark (v3.26) [33] was used to predict the coding regions (CDS) of the assembled contigs. The CDSs of all samples were clustered using CD-HIT (V4.6.1) to obtain unigenes.

### Taxonomy and function analysis

DIAMOND (v0.9.14) was applied to perform a taxonomic assessment of the gut microbiota based on the RefSeq database [34]. Microbial taxa with a relative abundance > 0.01% in more than 50% of the samples were used for downstream analysis. The abundance of Kyoto Encyclopedia of Genes and Genomes (KEGG) Orthology (KO) and pathways were normalized to transcripts per million (TPM) [35].

#### Fecal sample preparation, metabolite extraction, and identification of metabolomics analysis

Frozen feces (100 mg) were thoroughly ground with liquid nitrogen, mixed with 1 mL 50% methanol buffer, and incubated for 10 min. The mixture was stored at − 20 °C overnight to precipitate proteins and then centrifuged at 4000 × g for 20 min. The supernatants were used for liquid chromatography‒mass spectrometry (LC‒MS) analysis to detect metabolites. An ultra-performance liquid chromatography (UPLC) system (SCIEX, UK) equipped with an ACQUITY UPLC T3 column (100 mm × 2.1 mm, 1.8 μm, Waters, UK) was used for chromatographic reversed-phase separation. The TripleTOF 5600 Plus high-resolution tandem mass spectrometer (SCIEX, Warrington, UK) was operated in both positive and negative ion modes to detect metabolites eluted from the column. The TOF mass ranged from 60 to 1200 Da. XCMS software was used for the acquired MS data pretreatments and exported into the mzXML format [36].

### Metabolomics data processing

The LC‒MS raw data files were processed by XCMS, CAMERA [37], and MetaX toolbox in R for peak detection and the CAMERA package for peak annotation [38]. Each ion was identified by its retention time and m/z. The information with the matrix was mapped to public databases, including KEGG and BMDB (https://www.mdpi.com/2218-1989/10/6/23), with a threshold of 10 ppm [39]. MetaX was applied to filter peak intensity data with a standard that features should not be detected in less than 50% of QC samples. In addition, the relative standard deviations of the metabolic features were calculated across all QC samples, and standard deviations > 30% across all QC samples were removed. After quality control, the group datasets were normalized using the probabilistic quotient normalization algorithm and log transformed before analysis. The analysis of metabolite sources was performed in MetOrigin (2022–01 version; http://metorigin.met-bioinformatics.cn/app/metorigin) [40]. The metabolites were divided into four groups in MetOrigin, including the host group (metabolites only produced by the host), microbiota group (metabolites only produced by the microbiota), co-metabolism group (metabolites produced by both the host and microbiota), and others group, which consisted of drug-related, food-related, environmental, and unknown metabolites. And the significantly different metabolites were determined based on the variable *P* < 0.05 of Wilcoxon rank-sum test. The enrichment analysis included in MetOrigin was applied to each metabolite from each cluster to identify metabolic pathways (*P* < 0.05) [41].

### Fecal microbiota transplantation (FMT) in mice

For microbiota suspension preparation, we first defrosted the fecal samples of LOS and HOS cows that were stored at − 80 °C. Then, 5 g feces from donor cows in the LOS and HOS groups were dissolved in 50 mL physiological saline and mixed well. The fecal suspension was then passed through two gauze filters, the first with two layers and the second with four. This step was followed by a counting test on a blood cell counting plate to ensure that the concentration of microbes in the suspension was more than 10^8^ CFU/mL. Finally, the microbial suspensions from the LOS and HOS cows were mixed separately, 10% glycerin was added, and the samples were frozen at − 80 °C.

Forty-five female mice (C57BL/6 J) underwent a 7-day adaptation stage before treatment. Next, the mice were gavaged with a mixture of antibiotics (1 g/L ampicillin [#A7490, Solarbio], 0.5 g/L vancomycin [#Y25829, Yuanye], 0.5 g/L neomycin [#631307, Takara], and 1 g/L metronidazole) in 200 μL nuclease-free saline for 7 consecutive days to deplete the gut bacteria [42]. Subsequently, the mice were randomly divided into groups—CON, FLOS, and FHOS—which were orally gavaged with saline, fecal microbial suspension from LOS cows, or fecal microbial suspension from HOS cows, and the oral volume was 200 μL/d.

Fourteen days later, the mice were euthanized, and blood samples were collected by eyeball extirpating using blood collection tubes containing EDTA. Then, the blood samples were centrifuged at 1600 g/min at 4 °C for 12 min to obtain plasma for the determination of the parameters of OS, including T-AOC, TOS, OSI, MDA, SOD, CAT, GSH, and GSH-px. The methods used to determine these parameters were the same as those used for cows.

## Results

### The associations between fecal microbiota and postpartum oxidative stress

**Fig. 1:**
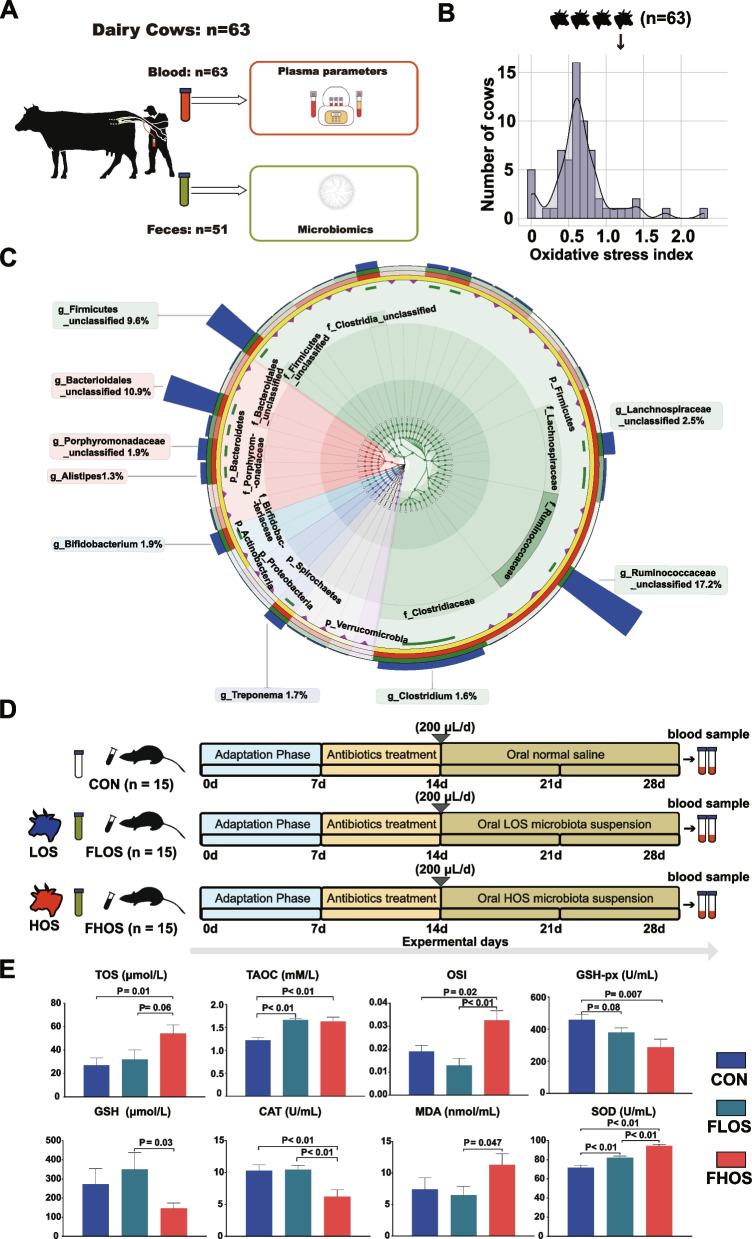
The overview of the oxidative stress and fecal microbial composition of postpartum dairy cows. **A** Study and sampling design of the cow trial. **B** The oxidative stress index profile of dairy cows at 7 days postpartum. **C** Taxonomic and phylogenetic trees of the gut microbiome by 16S rRNA gene sequencing. **D** Study design diagram of the fecal transplantation experiment. **E** Plasma oxidative stress parameters of mice in the four groups. LOS, cows with lower oxidative stress; HOS, cows with higher oxidative stress; CON, mice orally gavaged with saline; FLOS, mice orally gavaged with fecal microbial suspension from LOS cows; FHOS, mice orally gavaged with fecal microbial suspension from HOS cows. The *P* value < 0.10 were presented

A total of 63 healthy Chinese Holstein dairy cows at 7 days postpartum were selected for the collection of the blood and fecal samples (Fig. 1A). The plasma T-AOC and TOS of postpartum dairy cows ranged from 0.431 to 0.589 mM and from 0.000205 to 1.054 mM, respectively (Fig. S1A and B). The plasma OSI values ranged from 0.0008 to 2.3, with a mean value of 0.66 (Fig. 1B).

The retained reads of 16S sequencing ranged from 64,057 to 76,618 with high sampling coverage (> 95%) in all samples (Tables S2 and S3). At the phylum level, nine phyla were identified, and the dominant bacterial phyla included Firmicutes (37.7% ± 5.23%), Bacteroidetes (15.6% ± 3.14%), and Spirochaetes (1.73 ± 2.52%). The dominant bacterial genera were *unclassified Ruminococcaceae* (17.2 ± 3.33%), *unclassified Bacteroidetes* (10.9 ± 2.50%), *Firmicutes* (9.60 ± 1.74%), *Bifidobacterium* (1.90 ± 3.39%), and *Treponema* (1.70 ± 2.52%) (Fig. 1C and Table S4). The results of the LMM analysis of the data of 51 cows showed that 43.1% of the OSI variations could be explained by the gut microbial composition at the genus level.

To further investigate the direct effect of the gut microbiota on the host postpartum systemic OS, HOS, and LOS cows were selected. The OSI values were significantly higher (*P* < 0.01) in the HOS cows than in the LOS cows (1.02 ± 0.16 vs. 0.34 ± 0.07; Table 1). Plasma parameters were not significantly different between the two groups, except for GSH-px (*P* = 0.04) (Table 1). Additionally, the fecal microbiota from the HOS and LOS cows was transplanted into antibiotic-treated mice by oral gavage (Fig. 1D). The pseudosterile mouse model was successfully constructed based on the results of cecum morphology and fecal bacterial culture (Fig. S2A and B). No significant differences were observed in body weight among treatments (Fig. S2C). The results showed that the concentration of TOS in the FHOS group was significantly higher than that in CON (*P* = 0.01) and tended to be higher than that in the FLOS group (*P* = 0.06, Fig. 1E). The TAOC values were not significantly different between the FLOS and FHOS groups, but these values in the FLOS and FHOS groups were significantly higher than those in the CON group (*P* < 0.01). Notably, the OSI of the FHOS group was significantly higher than that of the CON (*P* = 0.02) and FLOS (*P* < 0.01) groups. Moreover, consistent with the cattle experiment, the GSH-px of the FHOS group was significantly lower than that of the CON group (*P* < 0.01). The GSH-px in the FLOS group tended to be lower than that in the CON group (*P* = 0.08). We also determined the plasma GSH concentration. We found that the GSH concentration was lowest in the FHOS group, and this concentration significantly differed from that of the FLOS group (*P* = 0.03). The SOD concentration in the FHOS group was higher than that in the CON and FLOS groups (*P* < 0.01); in contrast, the CAT content was significantly lower in the FHOS group (*P* < 0.01). Additionally, the mice in the FHOS group showed the highest concentration of MDA and that of the FHOS group was significantly higher than that of the FLOS group (*P* = 0.047) (Fig. 1E).

**Table 1:** Plasma physiological parameters, inflammation, oxidative stress, and phenotypic characteristics in dairy cows with low (LOS) and high oxidative stress (HOS)

Items	LOS	HOS	SEM	*P* value
**Physiological parameters**				
Alanine aminotransferase, U/L	17.8	17.6	1.46	0.92
Aspartate aminotransferase, U/L	109	110	12.0	0.93
Total protein, g/L	68.7	71.1	2.45	0.34
Albumin, g/L	31.9	34.0	1.07	0.07
Glucose, mM	3.31	3.64	0.21	0.13
Blood urea nitrogen, mM	4.48	3.95	0.50	0.31
Creatinine, μM	87.3	98.2	8.63	0.23
Cholesterol, mM	2.50	2.40	0.27	0.71
Triglyceride, mM	0.10	0.10	0.007	0.96
β-hydroxybutyrate, μM	862	1,020	162	0.33
Nonesterified fatty acid, μM	641	823	171	0.30
**Inflammation biomarkers**				
Haptoglobin, U/L	338	378	38.2	0.31
Serum amyloid A, μg/mL	36.7	39.6	3.08	0.36
**Oxidative stress biomarkers**				
Superoxide dismutase, U/mL	175	192	8.62	0.07
Total antioxidant capacity, mM	0.53	0.51	0.02	0.30
Catalase, U/mL	1.07	1.07	0.07	0.98
Glutathione peroxidase, U/mL	38.4	27.8	4.73	0.04
Malondialdehyde, nmol/mL	3.94	2.81	0.85	0.20
Total oxidative status, μM	183	517	80.8	< 0.01
Oxidative stress index^a^	0.34	1.02	0.18	< 0.01
**Phenotypic characteristics**				
Milk yield, kg/d	36.8	35.3	3.73	0.70
Parity	2.13	2.22	0.54	0.86
Body condition scores	2.67	2.96	0.25	0.26

^a^Oxidative stress index = total oxidative status/total antioxidant capacity

### Distinct microbial compositions between the HOS and LOS cows

**Fig. 2:**
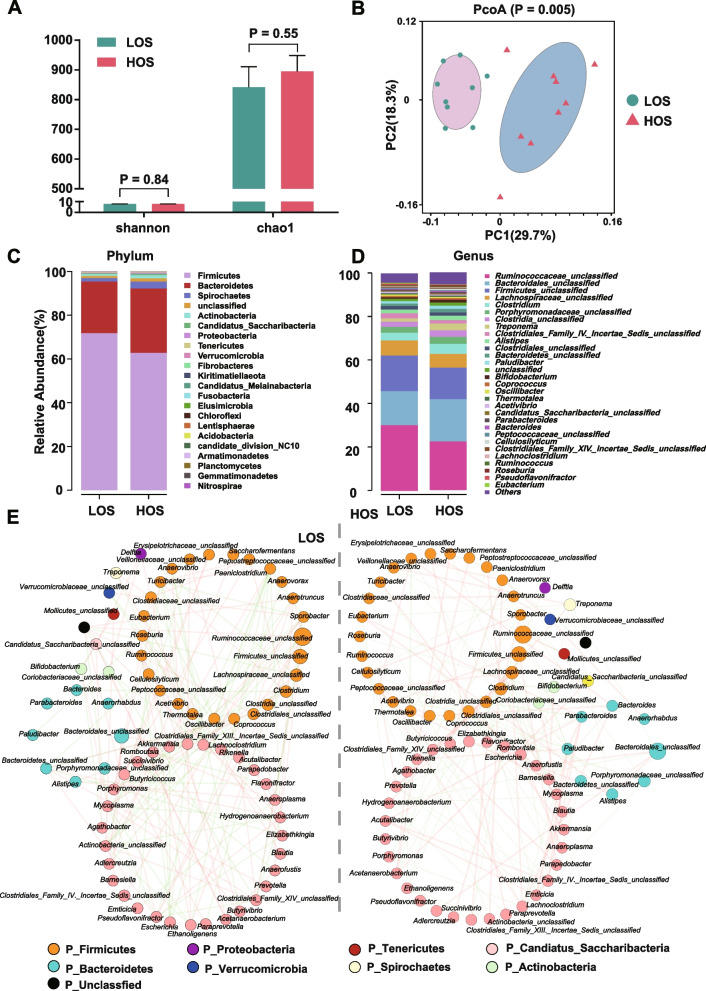
The difference in the fecal microbiome between cows with how (HOS) and low oxidative stress (LOS) according to the 16S rRNA gene sequencing data. **A** Changes in alpha diversity at the genus level. **B** Changes in beta diversity at the genus level. The *p* value was tested with ANOSIM. **C** Community biplot analysis at the family level. **D** Community biplot analysis at the genus level. **E** Genus co-occurrence network between LOS and HOS based on Spearman correlation analysis. Each node represents a bacterial genus; node size shows the relative abundance of each genus per group. The line refers to the Spearman coefficient. Red and green lines represent positive and negative interactions between nodes, respectively. Correlations with |rho|> 0.7 are presented

No significant differences in the Chao1 and Shannon indices of the fecal microbiome between the two groups were observed (Fig. 2A). However, principal coordinate analysis based on Bray‒Curtis showed distinct discrimination of microbial composition between the two groups at the genus level (ANOSIM* P* = 0.005; Fig. 2B). The bacterial taxa composition was similar at the phylum and genus levels between the LOS and HOS cows, but differences were observed in the relative abundance of the major phyla and genera between the two groups (Fig. 2C, D). The HOS and LOS cows mainly featured two co-occurrence networks with scattered genera from 8 phyla (Firmicutes, Bacteroidetes, Proteobacteria, Actinobacteria, Tenericutes, Verrucomicrobia, Spirochaetes, and Candiatus_Saccharibacteria) (Fig. 2E). The number of correlation edges (|rho|> 0.7) among the microbes in the HOS group was markedly lower than that in the LOS group, in which 147 and 125 of the edges were specific to the HOS and LOS cows, respectively. Four overlapping edges were shared between the two groups (Fig. S3A). The heatmap of closeness and the eigenvector of shared genera also presented the difference between the two groups (Fig. S3B).

**Fig. 3:**
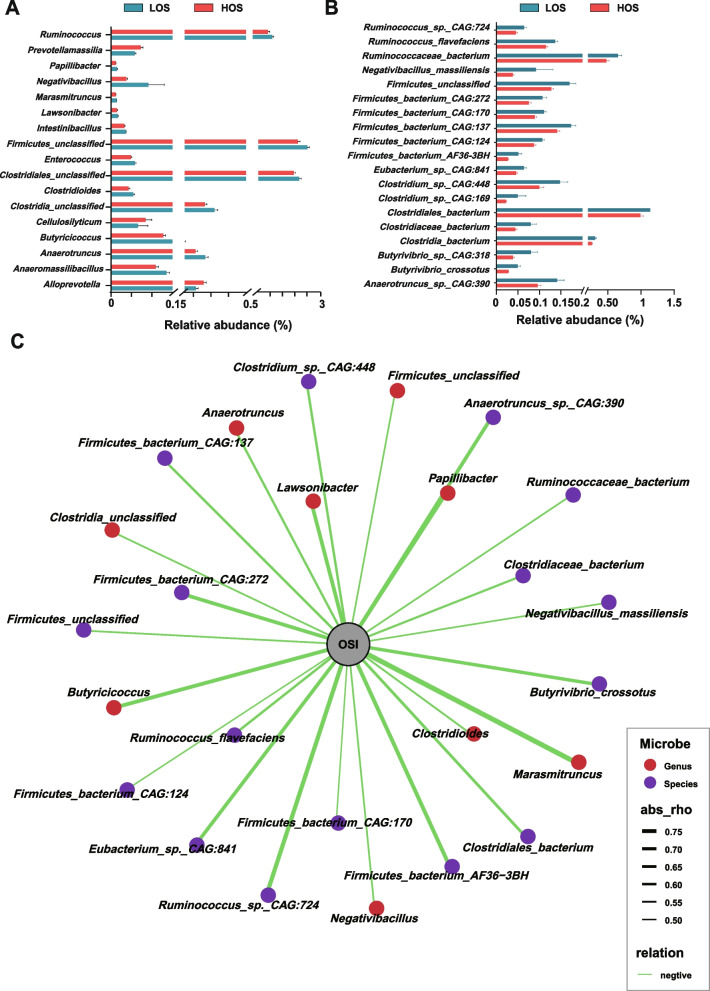
Gut microbiota divergence between cows with high (HOS) and low oxidative stress (LOS) at the species level based on metagenome sequencing data.** A** Abundance of significantly different bacterial genera between HOS and LOS. Significant differences were tested by linear discriminant analysis effect size analysis, with linear discriminant analysis (LDA) scores > 2 and a *P* value < 0.05. **B** Abundance of significantly different bacterial genera between HOS and LOS. Significant differences were tested by linear discriminant analysis effect size analysis, with linear discriminant analysis (LDA) scores > 2 and a *P* value < 0.05. **C** The network of the Spearman correlations between significantly different genera and species and plasma OSI between HOS and LOS cows. Interactions with a *P* value < 0.05 are presented

Metagenome sequencing generated 827,196,450 reads, with 45,955,358 ± 1,891,941 reads (mean ± SEM) per sample (Table S5). A total of 811,987,410 reads were retained, with 45,110,412 ± 1,887,842 reads per sample after quality control and removal of host genes. After de novo assembly, 4,207,139 contigs were generated (N50 length of 905 ± 56 bp), with 233,730 ± 54,483 contigs per sample. The gut fecal metagenome consisted of 90.0% bacteria, 0.679% archaea, 0.367% viruses, 0.0236% eukaryotes, and 8.9304% unclassified bacteria (Fig. S4A). A total of 1596 species were identified: 891 bacteria, 120 archaea, 231 viruses, 178 eukaryotes, and 176 unclassified species (Fig. S4B). LEfSe analysis identified 17 and 19 significantly different (*P* < 0.05, LDA > 2) bacterial genera and species between the two groups (Fig. 3A and B). The abundances of all the above taxa were significantly lower in the HOS cows, except for the genera *Prevotellamassilia*, *Cellulosilyticum*, and *Alloprevotella* (Fig. 3A). The correlation analysis results showed that most of these genera and species were significantly negatively correlated with the plasma OSI (Fig. 3A, Table S6), and among these the genus *Marasmitruncus* (Rho =  − 0.76, *P* = 0.0004) and species *Ruminococcus_sp._CAG:724* (Rho = -0.70, *P* = 0.002) showed the highest correlation with the OSI.

### Functional analysis of the metagenome and metabolome revealed lower GSH synthesis in the gut of HOS cows

**Fig. 4:**
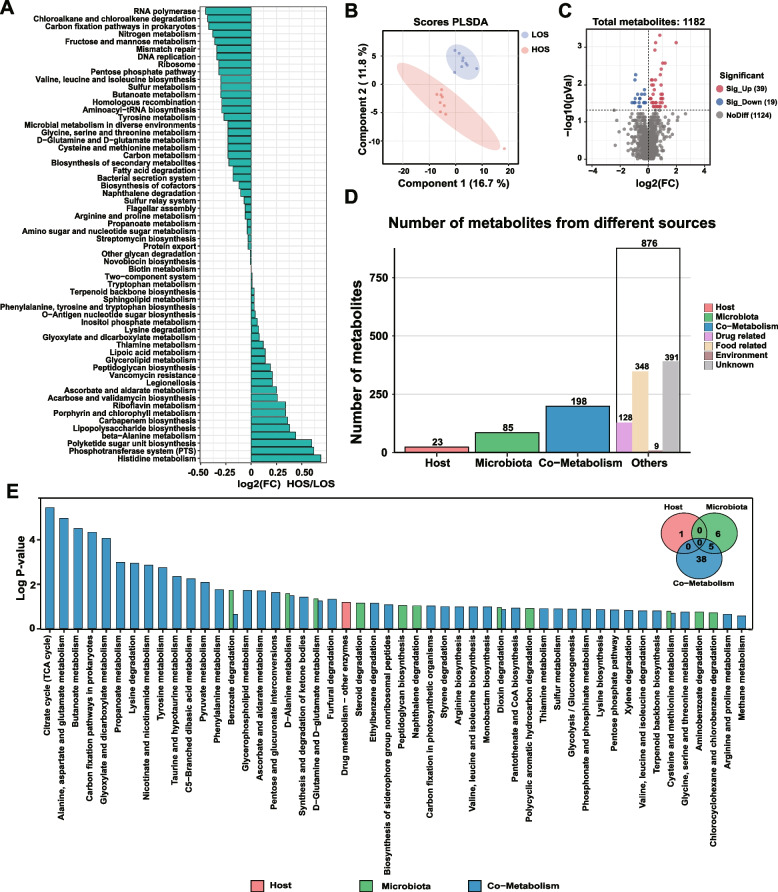
Differential KEGG functions of fecal microbiota between cows with high (HOS) and low oxidative stress (LOS). **A** Significantly different KEGG pathways of fecal microbiota between HOS and LOS; average transcripts per million of each pathway in HOS and LOS are presented. **B** Partial least squares-discriminant analysis of the fecal metabolome between HOS and LOS cows. **C** Volcano map of metabolites identified by the fecal metabolome. **D** Number of metabolites from different sources. **E** Metabolic pathway enrichment analysis according to different categories of metabolites belonging to the host, bacteria, or both

To explore the functional differences in fecal microbiota between HOS and LOS cows, KEGG enrichment analysis was performed with metagenome data. A total of 133 pathways were identified (Table S7). The significantly differential KEGG pathways are shown in Fig. 4A. There were 26 functional pathways enriched in HOS cows. Additionally, 35 functional pathways were enriched in LOS cows. Most of the differential pathways are associated with nitrogen metabolism, especially amino acid metabolism. Specifically, nitrogen metabolism (*P* = 0.05); D-glutamine and D-glutamate metabolism (*P* = 0.07); arginine and proline metabolism (*P* < 0.01); glycine, serine, and threonine metabolism (*P* < 0.03); and cysteine and methionine metabolism (*P* = 0.04) were all decreased in HOS cows.

Additionally, we performed fecal metabolome analysis for the HOS and LOS cows. The results of the partial least squares-discriminant analysis showed distinct clusters of metabolomes between the HOS and LOS cows (Fig. 4B). In total, 1182 compounds in feces were detected and quantified (Table S8), and 58 differential metabolites were identified (Fig. 4C and Table S9). Specifically, in the HOS group, the abundances of 39 metabolites were significantly lower, and the abundances of 19 metabolites were significantly higher than that in the LOS group (Fig. 4D). Furthermore, we identified the sources of the metabolites and found 23 metabolites from the host, 85 metabolites from the microbiota, 198 metabolites from host-microbiota cometabolism, and 876 metabolites from others, which consisted of drug (128), food (348), environment (9), and unknown (391) (Fig. 4D, Table S9). The abundances of 7 microbial metabolites, such as D-alanine (*P* = 0.0007, FC = 1.44), pyroglutamic acid (*P* = 0.007, FC = 1.42), and 1-aminocyclopropanecarboxylic acid (*P* = 0.01, FC = 1.28), were significantly higher in HOS cows (Table S9). In addition, a total of 12 metabolites from cometabolism showed significant differences between HOS and LOS cows; these metabolite include pyruvate (*P* = 0.003, FC = 2.29), succinic acid (*P* = 0.03, FC = 2.04) and oxoglutaric acid (*P* = 0.01, FC = 1.19). Next, we performed metabolic pathway enrichment analysis according to different categories of metabolites belonging to the host, bacteria, and both. A total of 55 pathways were identified, including 43 pathways from cometabolism, 11 from microbiota, and 1 from the host (Fig. 4E, Table S10). Additionally, the results showed that pathways, such as D-alanine metabolism (*P* = 0.009) and D-glutamine and D-glutamate metabolism (*P* = 0.0189), were enriched in microbiota or cometabolism and showed significant differences between the HOS and LOS cows. Additionally, cysteine and methionine metabolism (*P* = 0.10) and glycine, serine, and threonine metabolism (*P* = 0.10) tended to be different.

**Fig. 5:**
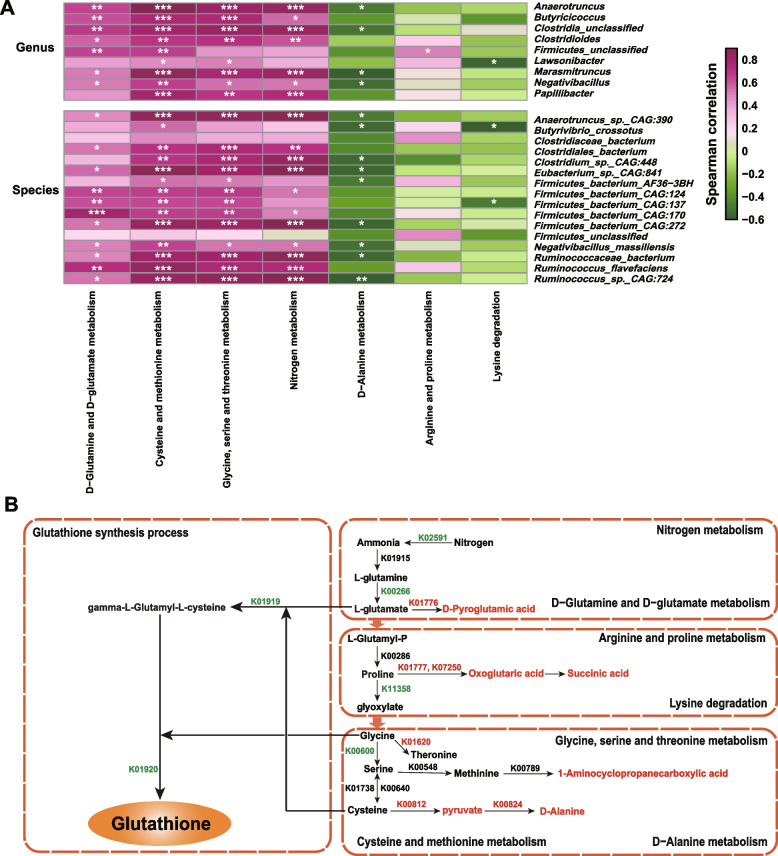
The integration analysis of the significantly differential microbes, microbial function, and metabolites. **A** The Spearman correlations between the significantly differential microbiota and the enriched metabolic pathways. The genera and species were selected from the significantly differential microbiota that were significantly correlated with oxidative stress status, and the pathways were enriched in the gut microbial functional analysis. *Represents the correlation *P* value < 0.05, **P value < 0.01, and ***P value < 0.001. **B** Integration of significantly different metabolic pathways involved in glutathione synthesis between HOS and LOS cows. KEGG Orthology (KO) entries with red and green words represent what was significantly increased and decreased in HOS compared with LOS, respectively, and black words indicate no significant difference observed between the two groups. The metabolites with red words represent the identified metabolites from microbiota or cometabolism by metabolome analysis and increased in the HOS cows. The names of the significant KO entries are as follows: K02591: nitrogenase molybdenum-iron protein beta chain [EC:1.18.6.1], K00266: glutamate synthase (NADPH) small chain [EC:1.4.1.13], K01776: glutamate racemase [EC:5.1.1.3], K01777: proline racemase [EC:5.1.1.4], K07250: 4-aminobutyrate aminotransferase/(S)-3-amino-2-methylpropionate transaminase/5-aminovalerate transaminase [EC:2.6.1.19 2.6.1.22 2.6.1.48], K01620: threonine aldolase [EC:4.1.2.48], K00600: glycine hydroxymethyltransferase [EC:2.1.2.1], K00812: aspartate aminotransferase [EC:2.6.1.1], and K00824: D-alanine transaminase [EC:2.6.1.21], K01919: glutamate–cysteine ligase [EC:6.3.2.2], K01920: glutathione synthase [EC:6.3.2.3], and K11358: aspartate aminotransferase [EC:2.6.1.1]

Consistent with these results, the correlation analysis showed that the significantly differential genera and species were significantly correlated with these pathways (Fig. 5A, Table S11), especially for D-glutamine and D-glutamate metabolism, cysteine and methionine metabolism, and glycine, serine, and threonine metabolism. Notably, all these pathways were involved in GSH synthesis (Fig. 5B). Moreover, the major enzymes and KO entries involved in the aforementioned KEGG pathways were also significantly different between the HOS and LOS cows (Fig. 5B and Table S12). For example, glutamate synthase (NADPH) small chain (K00266, *P* = 0.04) and glycine hydroxymethyltransferase (K00600, *P* = 0.04) were significantly lower, and glutamate-cysteine ligase (K01919, *P* = 0.10), glutathione synthase (K01920, *P* = 0.07), aspartate aminotransferase (K11358, *P* = 0.10), and nitrogenase molybdenum-iron protein beta chain (K02591, *P* = 0.09) tended to be lower in HOS cows. In contrast, glutamate racemase (K01776, *P* = 0.01), 4-aminobutyrate aminotransferase/(S)-3-amino-2-methylpropionate transaminase/5-aminovalerate transaminase (K07250, *P* = 0.03) and aspartate aminotransferase (K00812, *P* = 0.01) were significantly higher, and D-alanine transaminase (K00824, *P* = 0.09), proline racemase (K01777, *P* = 0.07), and threonine aldolase (K01620, *P* = 0.09) tended to be higher in the gut of HOS cows (Fig. 5B and Table S9). By integrating the changes between the two groups, including those in microbial metabolite abundance and the involved metabolic pathways, the results showed that fecal microbial GSH synthesis was lower in the gut of HOS cows than in that of LOS cows (Fig. 5B).

## Discussion

The alleviation of postpartum systemic OS in dairy cows is one of the biggest challenges to improving their health and welfare. The gut microbiota has attracted increasing attention owing to its underinvestigated effects on the physiological functions of the host [7]. Notably, in some diseases caused by a disturbed gut microbiome, the host OS increase is a possible pathogenic mechanism [8–10]. Here, we provided evidence that the fecal microbiota can contribute to host postpartum systemic OS by affecting GSH synthesis using multiomics approaches. This is the first study to reveal OS status-dependent changes in the fecal microbiota community of transition cows, and the results suggested that postpartum OS could also be a microbiome-linked pathology. This is attributed to the different microbial amino acid metabolism patterns involved in GSH synthesis.

Oxidative stress in transition cows is affected by various factors, such as different inflammation statuses, fat mobilization, and host genetics [43]. To estimate the proportions of variations in OSI caused by fecal microbial composition, we applied “microbiability,” which was first proposed by Difford et al. in dairy cows and calculated by quantifying the cumulative effects of microbial abundance on phenotypes [44]. This concept was inspired by the concept of biome-explainability, which is defined as the variance in host phenotype explained by the microbiome in a human study [45]. This method to date has been widely applied in animal studies [17, 46, 47]. The effect of the fecal microbiota on postpartum OS has not yet been reported in dairy cows. Compared with the effect of rumen microbiota on other traits, such as methane production (explainability is 13%) [44] and milk protein yield (explainability is 21.56%) [17], the explainability (43.1%) of the fecal microbiota to host OS is much higher. This result suggests that the fecal microbiota is strongly linked to host OS. The mice (FHOS) administered the microbiota of HOS cows showed a significantly higher OSI value, further confirming this hypothesis.

A number of significantly different genera and species between HOS and LOS cows were revealed in this study. The abundances of most of these genera and species were significantly lower in the HOS cows and negatively significantly correlated with OS. Moreover, the differential microbiota with larger abundance changes or greater correlations with OSI all belong to the members of Ruminococcaceae family bacterial taxa, such as *Negativibacillus* (with the greatest fold change), *Marasmitruncus*, and *Butyricicoccus* (with higher correlations with OSI). Previous studies have shown that the abundance of Ruminococcaceae serving as gut-beneficial bacteria was inversely correlated with some diseases accompanied by elevated OS [48–50]. Yang et al. reported that increased abundance of Ruminococcaceae could reduce hepatic OS in mice [51]. Consistent with these studies, Ruminococcaceae family bacterial taxa, such as *Ruminococcus* and *R. CAG:724*, *R. flavefaciens*, and *R. bacterium*, showed higher abundance in the LOS cows in this study. Additionally, *Negativibacillus* and *N. massiliensis* were reported to have a positive correlation with improved memory, which is accompanied by reduced OS of the host [52]. The increased abundance of *Butyricicoccus* was reported to alleviate oxidative status in mice [53]. Such results imply that changes in those microbiota may play important roles in host OS. Previous studies reported that the Ruminococcaceae family microbiota are mainly involved in nitrogen and amino acid metabolism [54, 55]. Therefore, the changes in the abundance of these microbiota may affect microbial amino acid metabolism and may be associated with the host OS level. In addition, the loss of gut microbial homeostasis can cause minor to severe nuisances at the local or systemic level, in turn, to OS [56]. Vernocchi et al. revealed that the reduced abundance of Clostridiaceae was associated with an imbalance in the gut microbiome [57]. Consistent with this, the lower abundance of Clostridiaceae bacteria, such as *C. bacterium*, *C. bacterium*, and *C. sp. CAG:448*, indicated that the HOS cows may had dysbiosis in gut microecology, which was supported by the results of the interacted correlation analysis among the microbes in the current study.

Despite the extensive attention given to the effects of the gut microbiota on host OS, there is a lack of understanding of the mechanisms underlying these outcomes. Here, we found that the different microbial metabolic patterns of amino acids, especially those involved in glutamine and glutamate metabolism, glycine metabolism, and cysteine metabolism, may contribute to host OS. Specifically, the fecal microbiota in LOS cows is more inclined to use glutamate, glycine, and cysteine to synthesize GSH, which plays a major role in the removal of many reactive species [58]. The higher abundance of enzymes, such as glutamate-cysteine ligase (K01919) and glutathione synthase (K01920), in LOS cows supported this finding. In addition, the results of metabolite source analysis further confirmed that the microbial amino acid metabolism pattern in HOS cows was different from that in LOS cows. In specific, glutamate was preferentially metabolized into pyroglutamic acid by glutamate racemase. However, cysteine was preferentially converted into pyruvate and alanine via transamination of aspartate aminotransferase and D-alanine transaminase in the fecal microbiota of HOS cows rather than synthesis to gamma-l-glutamyl-l-cysteine, which is the precursor of GSH. Consistent with this, GSH and GSH-px were significantly lower in FHOS mice than in CON and FLOS mice. Thus, these integrated results support the contribution of the fecal microbiota to host postpartum OS, which may be attributed to changes in microbial-mediated GSH synthesis. The regulatory molecular mechanisms require further research.

## Conclusions

The fecal microbiota is strongly associated with host postpartum OS, as changes in microbial composition, functions, and metabolites were different in OS dairy cows. The key microbial taxa included Ruminococcaceae family bacteria, such as *Negativibacillus*, *Marasmitruncus*, and *Butyricicoccus*. The lower GSH induced by fecal microbiota exerted essential roles in higher postpartum OS in transition dairy cows, and this can be attributed to the altered microbial amino acid metabolic patterns (e.g., glutamine, glutamate, glycine, and cysteine metabolism) involved in GSH synthesis. In light of our findings, targeted fecal microbiota and GSH synthesis-related amino acid metabolism regulatory strategies promise to provide unique insights into alleviating postpartum OS and improving the welfare and health of cows during the transition period.

## Supplementary Information

**Additional file 1: Table S1.** Ingredients of the basal diet (%, DM basis). **Table S2.** Summary of 16S rRNA gene sequencing data from fecal samples of 51 dairy cows. **Table S3.** The relative abundance of ASV generated by 16S rRNA sequencing in 51 cows. **Table S4.** The relative abundance of bactera in the feces of 51 cows at phylum, family and genus level. **Table S5.** Summary of sequence data generated from gut fecal samples of 9 LOS and 9 HOS cows. **Table S6.** The correlations of significantly the different microbiota and plasma OSI. **Table S7.** The renriched KEGG pathways (level3) in the gut of LOS cows and HOS cows. **Table S8.** The fecal metabolome profile of LOS and HOS cows. **Table S9.** The sources of the metabolites and differential analysis. **Table S10.** The enrichment analysis of the metabolites from microbiota, co-metabolism and host. **Table S11.** The correlation analysis between significant different microbiota and the major KEGG pathways.** Table S12.** Summary of identified KOEtries in the gut of LOS and HOS cows.

**Additional file 2: Fig. S1.** (A) The distribution of total oxidative status in 63 cows. (B) The distribution of total antioxidant capacity in 63 cows. **Fig. S2.** (A) The cecum morphology in before (7 d) and after antibiotic-treat (14 d) in mice. (B) The bacterial culture with feces of before (7 d) and after antibiotic-treated (14 d) mice. (C) The changes body weight during the fecal microbiota transplanting period. **Fig. S3.** (A) The number of unique and shared edges in co-occurrence networks of low (LOS) and high oxidative stress (HOS) cows. (B) The centralities (rank of the closeness) and discrepancies of nodes in LOS and HOS cows. **Fig. S4.** (A) Comparison of microbial domains between low (LOS) and high oxidative stress (HOS) cows. (B) The number of identified species in each domain.

## Data Availability

The 16S rRNA sequences of 51 cattle fecal samples and metagenome sequences of 18 cattle fecal samples were deposited into the NCBI Sequence Read Archive (SRA) under the accession numbers PRJNA786362 (https://dataview.ncbi.nlm.nih.gov/object/PRJNA786362?reviewer=uvvf8393625as0s947p8etat3a) and PRJNA786358 (https://dataview.ncbi.nlm.nih.gov/object/PRJNA786358?reviewer=7r7khodftc34ao1ff759fajd9l).

## References

[1] Ingvartsen KL (2006). Feeding and management-related diseases in the transition cow: physiological adaptations around calving and strategies to reduce feeding-related diseases. Anim Feed Sci Technol.

[2] Abuelo A, Hernández J, Benedito JL, Castillo C (2015). The importance of the oxidative status of dairy cattle in the periparturient period: revisiting antioxidant supplementation. J Anim Physiol Anim Nutr.

[3] Pascottini OB, Leroy JLMR, Opsomer G (2020). Metabolic stress in the transition period of dairy cows: focusing on the prepartum period. Animals.

[4] Sánchez-Rodríguez MA, Mendoza-Núñez VM (2019). Oxidative stress indexes for diagnosis of health or disease in humans. Oxid Med Cell Longev.

[5] Akkafa F, HalilAltiparmak I, Erkus ME, Aksoy N, Kaya C, Ozer A (2015). Reduced SIRT1 expression correlates with enhanced oxidative stress in compensated and decompensated heart failure. Redox Biol.

[6] Abuelo A, Hernández J, Benedito JL, Castillo C (2013). Oxidative stress index (OSi) as a new tool to assess redox status in dairy cattle during the transition period. Animal.

[7] Durack J, Lynch SV (2019). The gut microbiome: relationships with disease and opportunities for therapy. J Exp Med.

[8] Mohajeri MH, La Fata G, Steinert RE, Weber P (2018). Relationship between the gut microbiome and brain function. Nutr Rev.

[9] Li T, Zhang T, Gao H, Liu R, Gu M, Yang Y (2021). Tempol ameliorates polycystic ovary syndrome through attenuating intestinal oxidative stress and modulating of gut microbiota composition-serum metabolites interaction. Redox Biol.

[10] Petry AL, Huntley NF, Bedford MR, Patience JF (2020). Xylanase increased the energetic contribution of fiber and improved the oxidative status, gut barrier integrity, and growth performance of growing pigs fed insoluble corn-based fiber. J Anim Sci.

[11] Gong S, Feng Y, Zeng Y, Zhang H, Pan M, He F (2021). Gut microbiota accelerates cisplatin-induced acute liver injury associated with robust inflammation and oxidative stress in mice. J Transl Med.

[12] Tomasello G, Mazzola M, Leone A, Sinagra E, Zummo G, Farina F (2016). Nutrition, oxidative stress and intestinal dysbiosis: influence of diet on gut microbiota in inflammatory bowel diseases. Biomed Pap Med Fac Univ Palacky Olomouc Czech Repub.

[13] Dumitrescu L, Popescu-Olaru I, Cozma L, Tulbă D, Hinescu ME, Ceafalan LC (2018). Oxidative stress and the microbiota-gut-brain axis. Oxid Med Cell Longev.

[14] Bach A, López-García A, González-Recio O, Elcoso G, Fàbregas F, Chaucheyras-Durand F (2019). Changes in the rumen and colon microbiota and effects of live yeast dietary supplementation during the transition from the dry period to lactation of dairy cows. J Dairy Sci.

[15] Zalar B, Haslberger A, Peterlin B (2018). The role of microbiota in depression - a brief review. Psychiatr Danub.

[16] Yang H, Duan Z (2018). The local defender and functional mediator: gut microbiome. Digestion.

[17] Xue MY, Sun HZ, Wu XH, Liu JX, Guan LL (2020). Multi-omics reveals that the rumen microbiome and its metabolome together with the host metabolome contribute to individualized dairy cow performance. Microbiome.

[18] Subramanian I, Verma S, Kumar S, Jere A, Anamika K (2020). Multi-omics data integration, interpretation, and its application. Bioinform Biol Insights.

[19] Jiang D, Armour CR, Hu C, Mei M, Tian C, Sharpton TJ, Jiang Y (2019). Microbiome multi-omics network analysis: statistical considerations, limitations, and opportunities. Front Genet.

[20] Edmonson AJ, Lean IJ, Weaver LD, Farver T, Webster G (1989). A body condition scoring chart for holstein dairy cows. J Dairy Sci.

[21] Pertea G. fqtrim: v0.9.4 (Version 0.9.4). 2015. http://ccb.jhu.edu/software/fqtrim/index.shtml. Released 16 July 2015.

[22] Rognes T, Flouri T, Nichols B, Quince C, Mahé F (2016). VSEARCH: a versatile open source tool for metagenomics. PeerJ.

[23] Callahan BJ, McMurdie PJ, Rosen MJ, Han AW, Johnson AJ, Holmes SP (2016). DADA2: high-resolution sample inference from Illumina amplicon data. Nat Methods.

[24] Quast C, Pruesse E, Yilmaz P, Gerken J, Schweer T, Yarza P (2013). The SILVA ribosomal RNA gene database project: improved data processing and web-based tools. Nucleic Acids Res.

[25] Bolyen E, Rideout JR, Dillon MR, Bokulich NA, Abnet CC, Al-Ghalith GA (2019). Reproducible, interactive, scalable and extensible microbiome data science using QIIME 2. Nat Biotechnol.

[26] Segata N, Izard J, Waldron L, Gevers D, Miropolsky L, Garrett WS (2011). Metagenomic biomarker discovery and explanation. Genome Biol.

[27] Gu FF, Zhu SL, Tang YF, Liu XH, Jia MH, Malmuthuge N (2023). Gut microbiome is linked to functions of peripheral immune cells in transition cows during excessive lipolysis. Microbiome.

[28] Wang J, Zheng J, Shi W, Du N, Xu X, Zhang Y (2018). Dysbiosis of maternal and neonatal microbiota associated with gestational diabetes mellitus. Gut.

[29] Shannon P, Markiel A, Ozier O, Baliga NS, Wang JT, Ramage D (2003). Cytoscape: a software environment for integrated models of biomolecular interaction networks. Genome Res.

[30] Ziyatdinov A, Vázquez-Santiago M, Brunel H, Martinez-Perez A, Aschard H, Soria JM (2018). lme4qtl: linear mixed models with flexible covariance structure for genetic studies of related individuals. BMC Bioinformatics.

[31] Langmead B, Salzberg SL (2012). Fast gapped-read alignment with Bowtie 2. Nat Methods.

[32] Peng Y, Leung HC, Yiu SM, Chin FY (2012). IDBA-UD: a de novo assembler for single-cell and metagenomic sequencing data with highly uneven depth. Bioinformatics.

[33] Zhu W, Lomsadze A, Borodovsky M (2010). Ab initio gene identification in metagenomic sequences. Nucleic Acids Res.

[34] Buchfink B, Reuter K, Drost HG (2021). Sensitive protein alignments at tree-of-life scale using DIAMOND. Nat Methods.

[35] Wagner GP, Kin K, Lynch VJ (2012). Measurement of mRNA abundance using RNA-seq data: RPKM measure is inconsistent among samples. Theory Biosci.

[36] Gowda H, Ivanisevic J, Johnson CH, Kurczy ME, Benton HP, Rinehart D (2014). Interactive XCMS Online: simplifying advanced metabolomic data processing and subsequent statistical analyses. Anal Chem.

[37] Kuhl C, Tautenhahn R, Böttcher C, Larson TR, Neumann S (2012). CAMERA: an integrated strategy for compound spectra extraction and annotation of liquid chromatography/mass spectrometry data sets. Anal Chem.

[38] Wen B, Mei Z, Zeng C, Liu S (2017). metaX: a flexible and comprehensive software for processing metabolomics data. BMC Bioinformatics.

[39] Kanehisa M, Goto S, Kawashima S, Okuno Y, Hattori M (2004). The KEGG resource for deciphering the genome. Nucleic Acids Res.

[40] Yu G, CF Xu, DN Zhang, F Ju, Y Ni. MetOrigin: discriminating the origins of microbial metabolites for integrative analysis ofthe gut microbiome and metabolome. iMeta. 2022;e10. 10.1002/imt2.10. 10.1002/imt2.10 PMC10989983 38867728

[41] Xia J, Wishart DS (2010). MetPA: a web-based metabolomics tool for pathway analysis and visualization. Bioinformatics..

[42] Zeng SL, Li SZ, Xiao PT, Cai YY, Chu C, Chen BZ (2020). Citrus polymethoxyflavones attenuate metabolic syndrome by regulating gut microbiome and amino acid metabolism. Sci Adv.

[43] Sordillo LM, Raphael W (2013). Significance of metabolic stress, lipid mobilization, and inflammation on transition cow disorders. Vet Clin North Am Food Anim Pract.

[44] Difford GF, Plichta DR, Løvendahl P, Lassen J, Noel SJ, Højberg O (2018). Host genetics and the rumen microbiome jointly associate with methane emissions in dairy cows. PLoS Genet.

[45] Rothschild D, Weissbrod O, Barkan E, Kurilshikov A, Korem T, Zeevi D (2018). Environment dominates over host genetics in shaping human gut microbiota. Nature.

[46] Wen C, Yan W, Sun C, Ji C, Zhou Q, Zhang D (2019). The gut microbiota is largely independent of host genetics in regulating fat deposition in chickens. ISME J.

[47] Camarinha-Silva A, Maushammer M, Wellmann R, Vital M, Preuss S, Bennewitz J (2017). Host genome influence on gut microbial composition and microbial prediction of complex traits in pigs. Genetics.

[48] Bajaj JS, Heuman DM, Hylemon PB, Sanyal AJ, White MB, Monteith P (2014). Altered profile of human gut microbiome is associated with cirrhosis and its complications. J Hepatol.

[49] Bajaj JS, Hylemon PB, Ridlon JM, Heuman DM, Daita K, White MB (2012). Colonic mucosal microbiome differs from stool microbiome in cirrhosis and hepatic encephalopathy and is linked to cognition and inflammation. Am J Physiol Gastrointest Liver Physiol.

[50] Schnabl B, Brenner DA (2014). Interactions between the intestinal microbiome and liver diseases. Gastroenterology.

[51] Yang X, Mo W, Zheng C, Li W, Tang J, Wu X (2020). Alleviating effects of noni fruit polysaccharide on hepatic oxidative stress and inflammation in rats under a high-fat diet and its possible mechanisms. Food Funct.

[52] Sadovnikova IS, Gureev AP, Ignatyeva DA, Gryaznova MV, Chernyshova EV, Krutskikh EP (2021). Nrf2/ARE activators improve memory in aged mice via maintaining of mitochondrial quality control of brain and the modulation of gut microbiome. Pharmaceuticals.

[53] Zhang W, Zou G, Li B, Du X, Sun Z, Sun Y (2020). Fecal microbiota transplantation (FMT) alleviates experimental colitis in mice by gut microbiota regulation. J Microbiol Biotechnol.

[54] Wallis KF, Melnyk SB, Miousse IR (2020). Sex-specific effects of dietary methionine restriction on the intestinal microbiome. Nutrients.

[55] Ling CW, Miao Z, Xiao ML, Zhou H, Jiang Z, Fu Y (2021). The Association of gut microbiota with osteoporosis is mediated by amino acid metabolism: multiomics in a large cohort. J Clin Endocrinol Metab.

[56] Albenberg LG, Wu GD (2014). Diet and the intestinal microbiome: associations, functions, and implications for health and disease. Gastroenterology.

[57] Vernocchi P, Gili T, Conte F, Del Chierico F, Conta G, Miccheli A (2020). Network analysis of gut microbiome and metabolome to discover microbiota-linked biomarkers in patients affected by non-small cell lung cancer. Int J Mol Sci.

[58] Lu SC (1830). Glutathione synthesis. Biochim Biophys Acta.

