# Supplementary material for: The hindgut microbiome contributes to host oxidative stress in postpartum dairy cows by affecting glutathione synthesis process
Source: Microbiome. 2023 Apr 22;11:87. doi: 10.1186/s40168-023-01535-9 (PMC10122372; doi:10.1186/s40168-023-01535-9)
Supplement: Supplementary file 3 — Additional file 2: Fig. S1. (A) The distribution of total oxidative status in 63 cows. (B) The distribution of total antioxidant capacity in 63 cows. Fig. S2. (A) The cecum morphology in before (7 d) and after antibiotic-treat (14 d) in mice. (B) The bacterial culture with feces of before (7 d) and after antibiotic-treated (14 d) mice. (C) The changes body weight during the fecal microbiota transplanting period. Fig. S3. (A) The number of unique and shared edges in co-occurrence networks of low (LOS) and high oxidative stress (HOS) cows. (B) The centralities (rank of the closeness) and discrepancies of nodes in LOS and HOS cows. Fig. S4. (A) Comparison of microbial domains between low (LOS) and high oxidative stress (HOS) cows. (B) The number of identified species in each domain. [file 40168_2023_1535_MOESM2_ESM.docx]

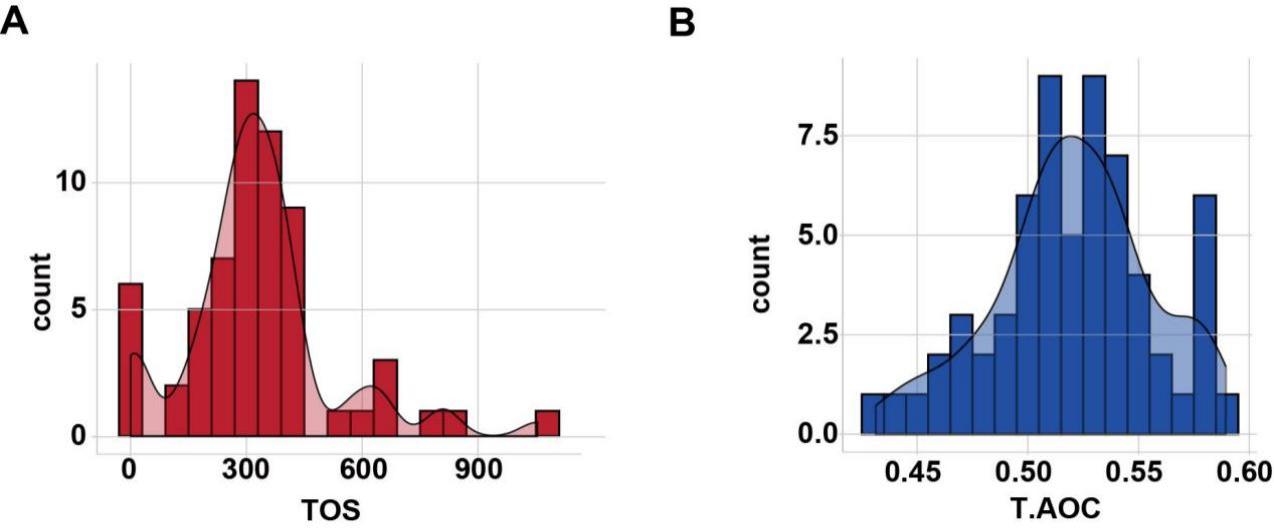


**Fig. S1.** (A) The distribution of total oxidative status in 63 cows. (B) The distribution of total antioxidant capacity in 63 cows.


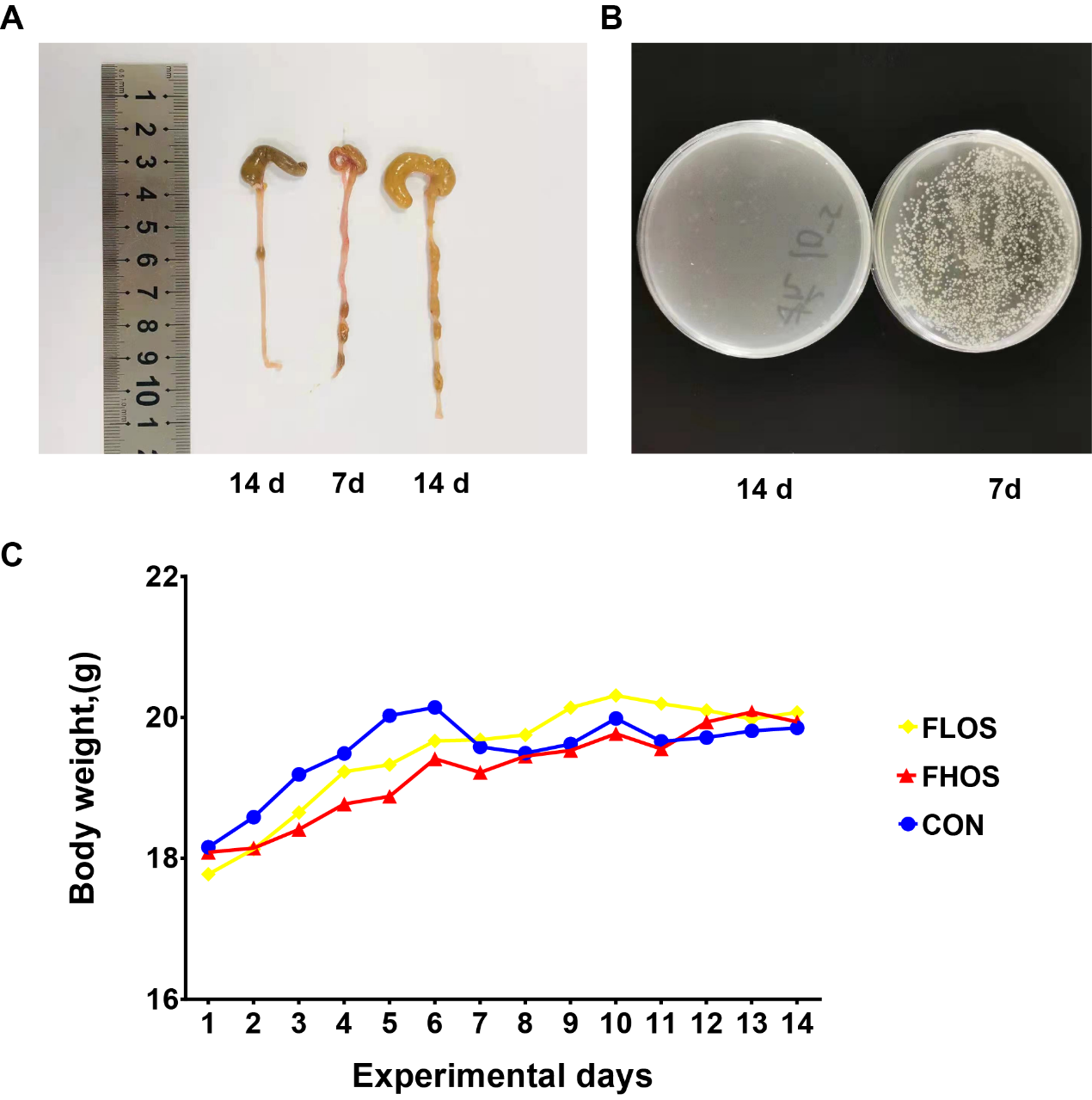


**Fig. S2.** (A) The cecum morphology in before (7 d) and after antibiotic-treat (14 d) in mice. (B) The bacterial culture with feces of before (7 d) and after antibiotic-treated (14 d) mice. (C) The changes body weight during the fecal microbiota transplanting period.


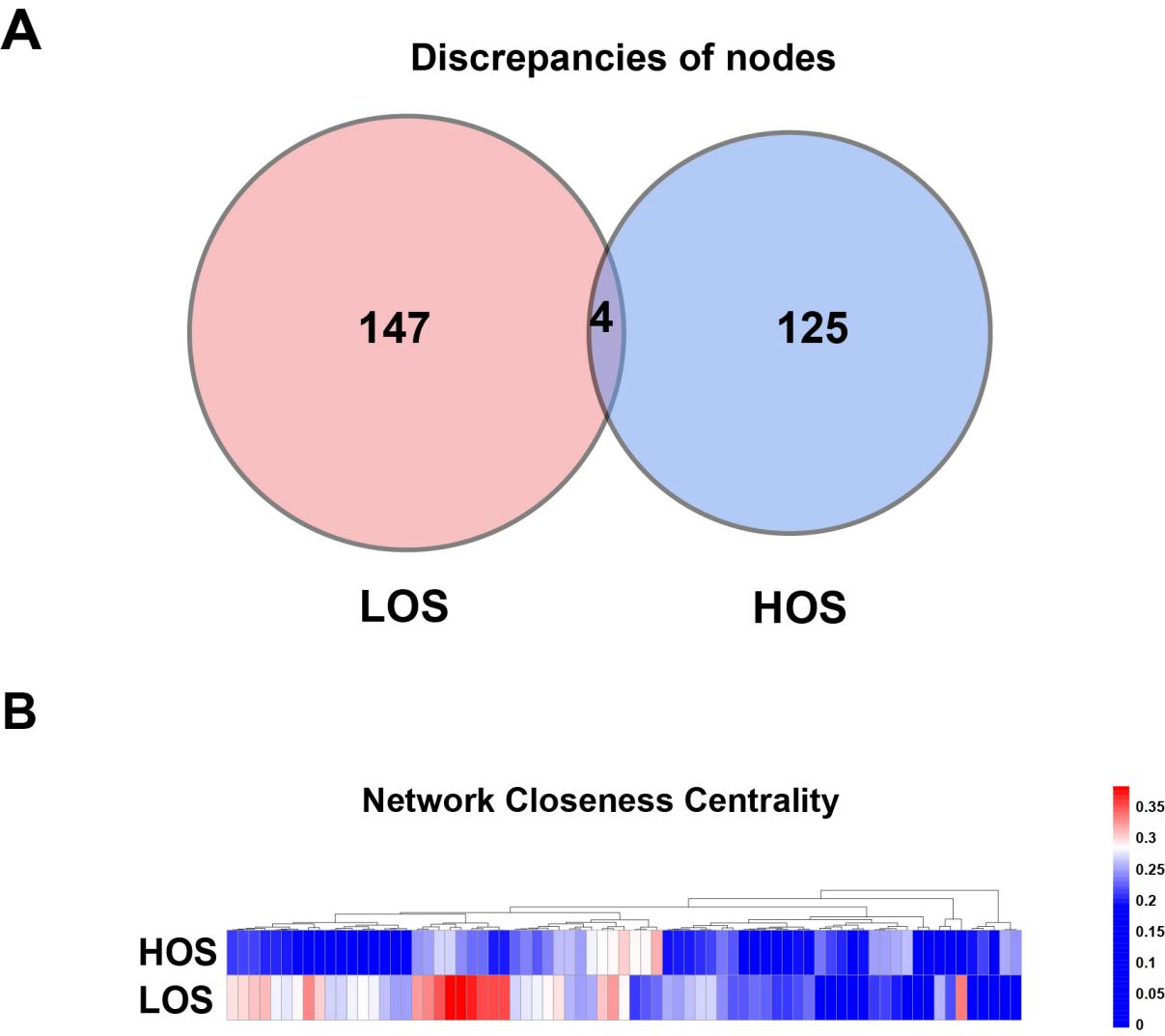


**Fig. S3.** (A) The number of unique and shared edges in co-occurrence networks of low (LOS) and high oxidative stress (HOS) cows. (B) The centralities (rank of the closeness) and discrepancies of nodes in LOS and HOS cows.


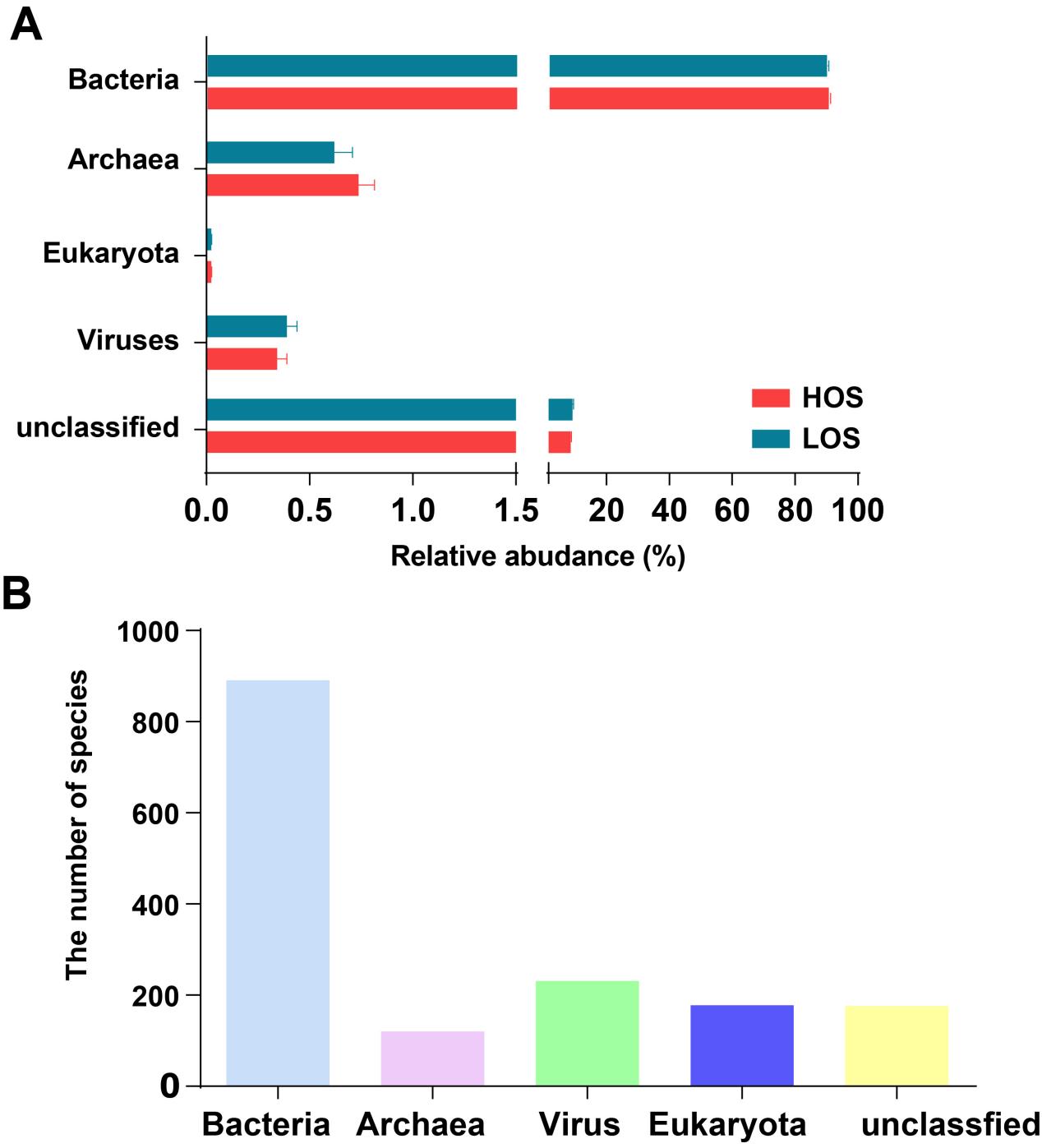


**Fig. S4.** (A) Comparison of microbial domains between low (LOS) and high oxidative stress (HOS) cows. (B) The number of identified species in each domain.
